# ZnO/ZnAl_2_O_4_ Nanocomposite with 3D Sphere-Like Hierarchical Structure for Photocatalytic Reduction of Aqueous Cr(VI)

**DOI:** 10.3390/ma11091624

**Published:** 2018-09-05

**Authors:** Xiaoya Yuan, Xin Cheng, Qiuye Jing, Jiawei Niu, Dong Peng, Zijuan Feng, Xue Wu

**Affiliations:** College of Materials Science and Engineering, Chongqing Jiaotong University, Chongqing 400074, China; cx1241513800@163.com (X.C.); m15922871980@163.com (Q.J.); Niujw2018@163.com (J.N.); pd2018@126.com (D.P.); fzj616@126.com (Z.F.); snowly199303@163.com (X.W.)

**Keywords:** hierarchical, ZnO, ZnAl_2_O_4_, photoreduction, Chromium(VI), UV light, nanomaterials

## Abstract

Three dimensional (3D) ZnO/ZnAl_2_O_4_ nanocomposites (Zn_n_Al-MMO) were synthesized by a simple urea-assisted hydrothermal process and subsequent high-temperature calcination. The as-prepared samples and their precursors were characterized by X-ray diffraction (XRD), Scanning electron microscopy (SEM), Transmission electron microscopy (TEM), UV-Vis diffuse reflectance spectroscopy (DRS), and Photoluminescence spectra (PL). It was observed that the morphology of Zn_n_Al-MMO nanocomposites could be tuned from cubic aggregates, hierarchically flower-like spheres to porous microspheres by simply changing the molar ratio of metal cations of the starting reaction mixtures. The photocatalytic performance of ZnO/ZnAl_2_O_4_ nanocomposites in the photoreduction of aqueous Cr(VI) indicated that the as-prepared 3D hierarchical sphere-like Zn_n_Al-MMO nanocomposite showed excellent photocatalytic activity of Cr(VI) reduction under UV light irradiation. The results indicated that the maximum removal percentage of aqueous Cr(VI) was 98% within four hours at 10 mg/L initial concentration of Cr(VI), owing to the effective charge separation and diversion of photogenerated carriers across the heterojunction interface of the composite. Our study put forward a facile method to fabricate hierarchical ZnO/ZnAl_2_O_4_ composites with potential applications for wastewater treatment.

## 1. Introduction

Chromium is common environmental pollutant and it is listed as a priority pollutant by the U.S. Environmental Protection Agency (EPA) [1], which mainly originates from industrial processes, such as leather tanning, metallurgy, electroplating, rubber, and ceramics [2]. Chromium-containing wastewater is currently recognized as one of the most serious environmental hazards, mainly occurring in two common oxidation states of Cr(III) and Cr(VI) in water. The Cr(VI) species are known to be highly toxic and carcinogenic, whose toxicity is one hundred times than that of Cr(III) [3,4]. Therefore, how to dispose of Cr(VI)-containing wastewater prior to its discharge is an important research topic of environmental protection and comprehensive utilization. The removal of Cr(VI) from the aquatic environment has been extensively investigated and it can be achieved by means of many techniques, such as cross flow microfiltration [5], chemical reduction precipitation [6], adsorption [7], ion exchange [8], electrolysis [9], reverse osmosis [10], photocatalysis technology [11], and so on. Among them, photoreduction technology of aqueous Cr(VI) is considered to be a promising method due to its simplicity of operation, high efficiency, and the inexhaustible solar energy. 

ZnO as a semiconductor photocatalyst has been widely studied for its advantages, such as high efficiency, non-toxicity, and low cost. Nevertheless, ZnO as a wide band gap (E_g_) semiconductor (3.37 eV) sustains an inherent limitation to only utilize UV light about 4% of solar energy for photoexcitation in the photocatalytic process. Adjusting the ZnO band structure to the lower band gap to accommodate light absorption is one of the main strategies. Therefore, the ZnO-based semiconductor composites have attracted tremendous research interests. The nanocomposites composed of N-doped ZnO/g-C_3_N_4_ core-shell nanosheets were prepared for the photocatalytic degradation of rhodamine B with visible-light and the results demonstrated that the photocatalytic removal rate of N-doped ZnO/g-C_3_N_4_ was more than six times higher than that of pure ZnO and g-C_3_N_4_ [12]. Arin et al. [13] used microwave irradiation to prepare ZnO/TiO_2_ nanocomposites and discussed their photocatalytic degradation of organic dye methylene blue in UV light with very high efficiency.

Moreover, ZnO has a rich nanostructures with differences in shape, size, and arrangement for different properties, including plates, pins, wires, ribbons, rods, balls, and so on [14,15,16,17]. Especially, hierarchical nanostructures have become an attractive class of materials due to their special structure and distinct properties [18,19,20,21]. Three-dimensional (3D) hierarchical structures are assembled from one-dimensional (1D) and two-dimensional (2D) nano-scale building blocks. So far, functional materials in various hierarchical structures can offer more opportunities to explore novel properties and superior device performances [22,23,24]. This structure is usually associated with a loose structure and large surface area, both characteristics of which can provide more active sites, while the hierarchical structure provides advantageous transmission pathways for electrons and holes, respectively, which can result in performances that are superior to congeneric nanomaterials [25,26]. For example, photocatalytic activities can be enhanced in the degradation of organic pollutants by hierarchical structure due to its unique structure [25,27,28].

Zinc aluminate (ZnAl_2_O_4_) is also extensively-studied semiconductor and its photocatalytic property has attracted the attention of researchers [29,30]. The ZnO/ZnAl_2_O_4_ composite photocatalyst not only has excellent stability, but it also has a much higher photocatalytic activity than a single oxide. Zhang et al. [31] prepared ZnO/ZnAl_2_O_4_ composite with hollow microspheres by hydrothermal process and the maximum photocatalytic decoloration rate of methyl orange was 98.7% within 60 min under the photocatalyst concentration of 0.5 g/L. Zhao et al. [32] fabricated a series of ZnO/ZnAl_2_O_4_ nanocomposites as plate-like by heated treatment of ZnAl-layered double hydroxide (ZnAl-LDH) precursors with different Zn/Al molar ratios at 800 °C in order to evaluate the photodegradation performance of methyl orange dye under artificial ultraviolet light. According to the survey, there are few studies involving the photocatalytic reduction of aqueous Cr(VI) while using hierarchical-structured ZnO-based photocatalysts are reported.

Herein, we reported a pacific preparation of ZnO/ZnAl_2_O_4_ nanocomposites with 3D sphere-like hierarchical structure via urea hydrothermal process and subsequent calcination at 900 °C for 4 h. The morphology of ZnO/ZnAl_2_O_4_ nanocomposites was tuned from cubic aggregates, hierarchically flower-like spheres to porous microspheres by simply changing the molar ratio of metal cations of the initial reaction mixtures. The photocatalytic activities of these nanocomposites were further evaluated via the photoreduction of aqueous Cr(VI) under UV light irradiation and it was found that only ZnO/ZnAl_2_O_4_ nanocomposites with 3D hierarchical sphere-like structure exhibited perfect photocatalytic activity for Cr(VI) reduction, owing to the effective charge separation and the diversion of photogenerated carriers across the interface of the composite.

## 2. Experimental Section

### 2.1. Synthesis of ZnO/ZnAl_2_O_4_ Nanocomposite

All the chemicals used in this study were of analytical grade and they could be used without further purification. Deionized (DI) water was used in all experiments.

The Zn_n_Al precursors were synthesized while using urea hydrothermal method and n value referred to the Zn to Al molar ratio of the starting mixture, which were 5.0, 7.0, and 9.0. A mixture of Zn(NO_3_)_2_·6H_2_O and Al(NO_3_)_3_·9H_2_O in different Zn/Al molar ratios were dissolved in DI water to obtain a solution with a total cationic concentration of 0.04 M, that is, Zn^2+^ + Al^3+^ = 0.04 M. While maintaining the molar ratio of (Zn^2+^ + Al^3+^)/urea = 1:5, urea was added to the nitrates solution. Then, the clear aqueous solutions were transferred to a 100 mL Teflon-lined reactor and maintained at 150 °C for 24 h. The white precipitates were collected and rinsed three times with DI water until pH = 7 after cooling to room temperature. Finally, the white precipitates were dried in an oven at 80 °C for 12 h. The ZnO/ZnAl_2_O_4_ nanocomposites were obtained by calcining the synthesized Zn_n_Al precursors in air at 900 °C for 4 h and the prepared nanocomposites were denoted as Zn_5_Al-MMO, Zn_7_Al-MMO, and Zn_9_Al-MMO, respectively.

### 2.2. Characterization

XRD patterns were collected in a PANalytical X’pert Pro powder diffractometer (PANalytical, Almelo, Netherlands) through Cu-Ka radiation (λ = 1.5418 Å) with a scan step of 0.013°. SEM (Hitachi, Tokyo, Chiyoda-ku, Japan) was used to study the morphology of some selected samples through a Hitachi SU8020 scanning electron microscope with acceleration voltage of 20 kV. HRTEM images were obtained by a FEI Tecnai G2 F20 field-emission transmission electron microscopy (FEI Inc., Hillsboro, OR, USA) at an accelerating voltage of 200 kV. Diffuse reflectance spectroscopy (DRS) was performed on a Hitachi U-4100 UV-vis spectrophotometer (Hitachi, Tokyo, Chiyoda-ku, Japan) while using BaSO_4_ as the reference sample. Room temperature PL measurements were done using a Hitachi F-7000 fluorescence spectrophotometer (Hitachi, Tokyo, Chiyoda-ku, Japan).

### 2.3. Photocatalytic Performance Tests

The effect of Zn_n_Al-MMO on the photocatalytic reduction performance of aqueous Cr(VI) was implemented using a 500 W high-pressure Hg lamp (BILON-CHX-V, Shanghai photoreactor, BiLon, Shanghai, China), with a maximum wavelength emission of 365 nm. Stock solution (150 mg/L) of Cr(VI) was prepared by dissolving K_2_Cr_2_O_7_ in distilled water and was diluted to the desired concentration prior to use. In each runs, 200 mg photocatalyst was dispersed in 50 mL of different concentrations of aqueous Cr(VI) solutions. Before irradiation, the mixture solution was magnetically stirred in the dark for 60 min to allow for the adsorption-desorption equilibrium to be established. While keeping magnetic stirring, 4 mL of the reaction product was extracted at regular intervals during the irradiation and the photocatalyst were separated by centrifugation from the solution. Determination of Cr(VI) concentration in the supernatant by UV-1000 spectrophotometer (AOE, Shanghai, China) at 540 nm from 1,5-diphenylcarbazide method [33]. As a comparison, the blank experiment was carried out under UV irradiation to monitor the stability of Cr(VI) and the dark experiments to monitor the physical adsorption capacity of Zn_n_Al-MMO photocatalyst under the same conditions.

## 3. Results and Discussion

### 3.1. Characterization of Zn_n_Al Precursors and Zn_n_Al-MMO Nanocomposites

**Figure 1:**
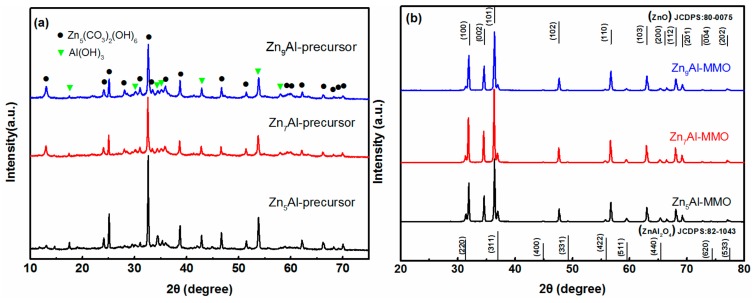
XRD patterns of (**a**) Zn_n_Al precursor and (**b**) Zinc aluminate nanocomposites (Zn_n_Al-MMO) with different Zn/Al molar ratio.

Figure 1 shows the XRD patterns of Zn_n_Al precursors and Zn_n_Al-MMO nanocomposites. For Zn_n_Al precursor, the characteristic reflections at 2θ = 13.4, 24.2, 28.3, 32.7, 54.2, 58.6, and 64.5° were observed, which were attributed to (200), (310), (020), (021), (800), (−622), and (−532) planes of the typical well-crystalized Zn_5_(CO_3_)_2_(OH)_6_ materials (JCPDS: 19-1458). The remain diffraction peaks donated with “▼” came from Al(OH)_3_ (JCPDS: 33-0018). The result showed that the precursors mainly consisted of Zn_5_(CO_3_)_2_(OH)_6_ and Al(OH)_3_ phases. As shown in Figure 1b, the XRD results indicated that all of the as-prepared powder samples became polycrystalline after the high-temperature calcination of the precursors. The diffraction peaks coincided with the mixed-phases of hexagonal ZnO (JCPDS: 80-0075) matching with standard diffraction data 31.8°(100), 34.4°(002), 36.2°(101), 47.4°(102), 56.5°(110), 62.8°(103), 67.9°(112), and the face-centered cubic spinel-structured ZnAl_2_O_4_ (JCPDS: 82-1043) at reflections 31.4°(220), 36.9°(311), 44.8°(400), 49.1°(331), 55.6°(422), and 65.2°(440). Meanwhile, the diffraction reflections of ZnO phase were much sharper and stronger than those of ZnAl_2_O_4_, indicating that ZnO phase had high crystallinity and dominance in MMO, which was beneficial to its photocatalytic activity in Cr(VI) photoreduction.

**Figure 2:**
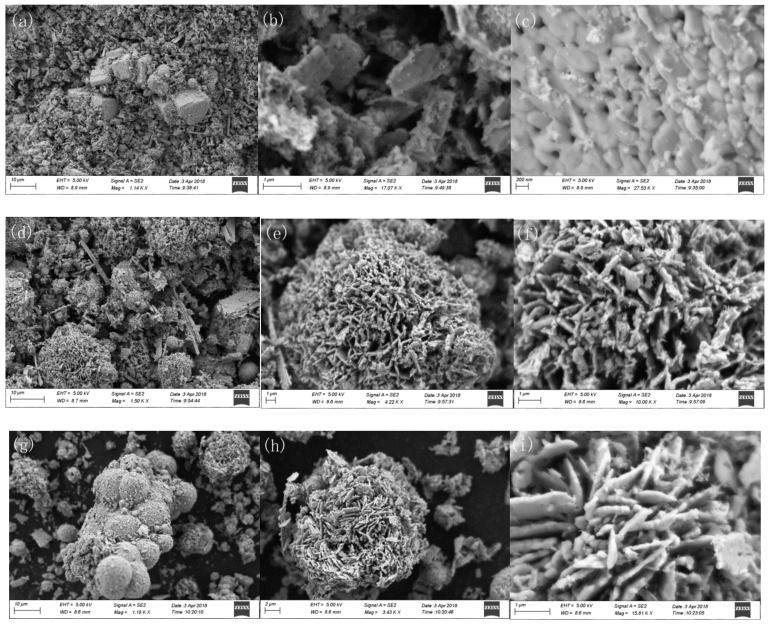
SEM images for (**a**–**c**) Zn_5_Al-MMO, (**d**–**f**) Zn_7_Al-MMO, and (**g**–**i**) Zn_9_Al-MMO.

The morphologies of the as-prepared Zn_n_Al-MMO nanocomposite were investigated by SEM. As shown in Figure 2a,b, Zn_5_Al-MMO displayed irregular flake-morphology and many cubic aggregates made up of irregular crystallites were visible on their surfaces. Figure 2c presents that these irregular amorphous nanoplates had a relatively rough surface and many pores were also detected, resulting from the release of CO_2_ during the calcination of Zn_5_(CO_3_)_2_(OH)_6_ at high temperature. From SEM images of Figure 2d–f, the sample Zn_7_Al-MMO was found to be consisted of many 3D flower-like spheres with a diameter of around 3–23 μm. These uneven assemblies were composed of dozens of closely impacted irregular nanoplatelets with thickness below 250 nm (Figure 2f). Notably, plenty of nano-scale pores between the oriented inter-connected platelets were also obviously observed, which contributed to provide more pollutant adsorption sites and harvest the solar light. The Zn_9_Al-MMO sample was prepared under the identical reaction conditions except with higher Zn^2+^ to Al^3+^ molar ratio in the starting nitrate mixture. In this case, more monodispersable porous assemblies (Figure 2g) and hierarchically 3D flower-like spheres morphology (Figure 2h) were observed. The diameter of porous microspheres was in the range of 7–14 μm. When compared to that of the Zn_7_Al-MMO sample, Zn_9_Al-MMO appeared smaller in diameter and the oriented nanosheets in the microspheres were thicker. Moreover, the assembled-structure of Zn_7_Al-MMO was much looser than that of Zn_9_Al-MMO. Thus Zn_7_Al-MMO was expected to provide more catalytic sites on the surface than Zn_9_Al-MMO. The SEM results revealed that the morphologies of the prepared Zn_n_Al-MMO nanocomposites, from cubic aggregates, porous microspheres to hierarchically 3D flower-like spheres, highly relied on the molar ratio of Zn/Al metallic cations of the starting materials. Huo reported that hierarchical ZnO/ZnAl_2_O_4_ composite that was synthesized by calcining ZnAl-LDH at 800 °C revealed outstanding photocatalytic activity in degrading methylene blue to bare ZnO phase and they ascribed the hierarchical nanostructure and high specific surface areas of ZnO/ZnAl_2_O_4_ composite to the superior photocatalytic performance [34]. It is reasonable to conclude that the hierarchically 3D sphere-like structure of Zn_n_Al-MMO could be beneficial to photocatalysis based on our high Cr(VI) photoredution activity (see Section 3.2 below).

**Figure 3:**
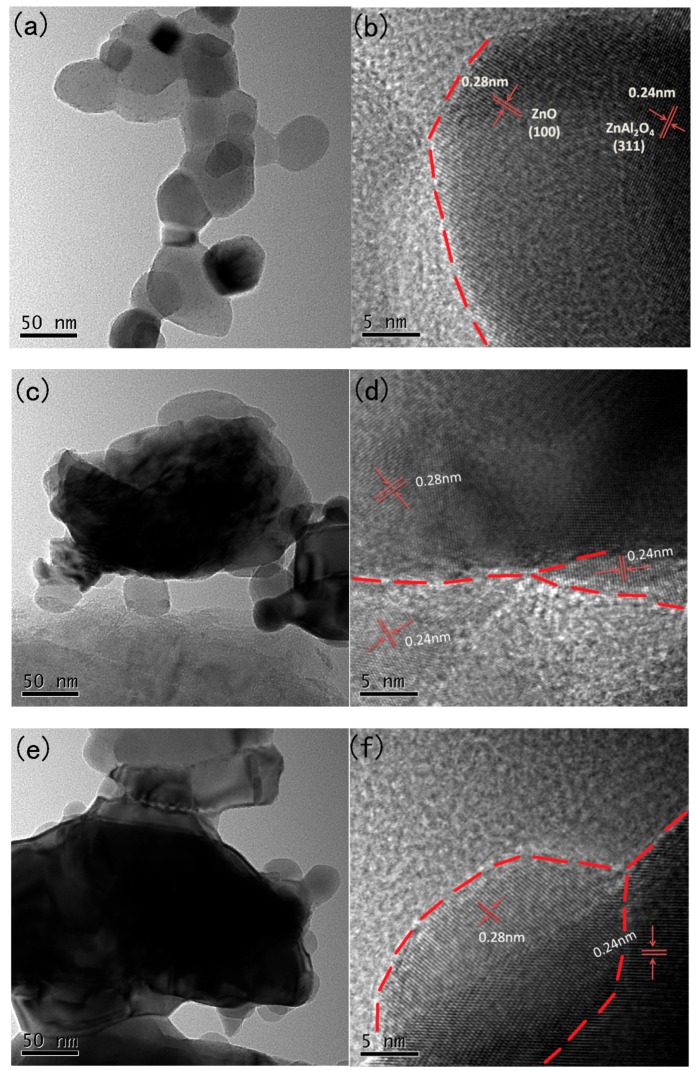
HRTEM images of (**a,b**) Zn_5_Al-MMO, (**c,d**) Zn_7_Al-MMO, and (**e,f**) Zn_9_Al-MMO.

More details about structural information of the oriented-nanosheets composing Zn_n_Al-MMO were provided by HRTEM studies (Figure 3). From Figure 3a, some hexagonal nanoplatelets and irregular nanosquares were detected, which was assigned to hexagonal ZnO and cubic ZnAl_2_O_4_ with lateral dimensions of 30–100 nm, respectively. Enlarged HRTEM images of Figure 3b revealed that oriented nanoplatelets was highly crystallized, showing two types of lattice fringes. The lattice fringes with an equal spacing of 0.28 nm matched well with the (100) planes of pure wurtzite ZnO phase [35] and 0.24 nm corresponded to the (311) facets of ZnAl_2_O_4_ crystal [36], further confirming that the oriented-nanosheets of Zn_5_Al-MMO aggregates consisted of ZnO and ZnAl_2_O_4_ crystal phases. This result was in line with the XRD analysis that is presented in Figure 1. From HREM images of Zn_7_Al-MMO and Zn_9_Al-MMO composites, either ZnO or ZnAl_2_O_4_ as the building blocks of the nanoplatelets in hierarchically 3D flower-like microspheres were not easily detected (Figure 3c,e), suggesting that strong interaction existed between the inter-connected nanoplatelets because microwave was employed for the HRTEM examination. Two lattice fringes were also observed as those for Zn_5_Al-MMO, indicating high crystallinity in all of the samples (Figure 3d,f). Additionally, the interface between ZnO and ZnAl_2_O_4_ phases in all MMO nanoflakes was easily observed from Figure 3b,d,f, which was formed by the sintering effect of ZnAl_2_O_4_ nanoparticles grown on the continuous ZnO phase [32]. Similar heterojunction has been found in lots of semiconductor-based photocatalyst composites [37], and is known to preferably promote photo-generated carriers separation in photocatalytic process [38,39,40].

**Figure 4:**
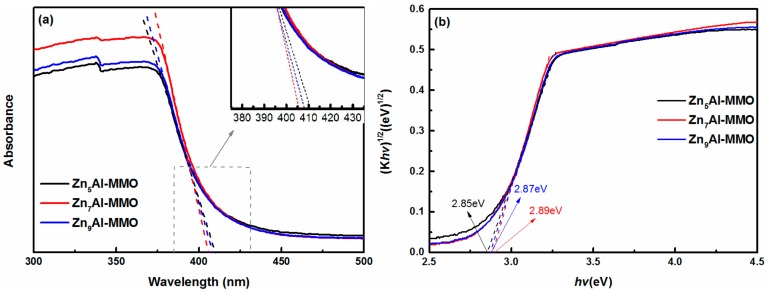
(**a**) Diffuse reflectance spectroscopy (DRS) spectra and (**b**) plots of (*k*h*v*)^1/2^ versus h*v* of Zn_n_Al-MMO composites with different Zn/Al molar ratios.

Figure 4a shows DRS spectra of Zn_n_Al-MMO composites with different Zn to Al molar ratio. The spectra revealed that there was strong absorption between 200–400 nm (ultraviolet region), while 400–800 nm (visible area) has almost no absorption for these three samples. As Zn^2+^ to Al^3+^ molar ratio increased, MMO appeared slightly blue-shifted adsorption edge and the absorption intensity was observed to increased and then decreased. Zn_7_Al-MMO nanocomposite has the highest adsorption intensity among all the three samples, which could be ascribed to the 3D sphere-like hierarchical and loose structure.

Figure 4b calculated the band gap energy (E_g_) by the DRS spectra. The E_g_ were determined by plotting graphs of (*K*h*ν*)^1/2^ versus (h*v*), with the help of reflectance spectra. The extrapolation of linear region of this plot to (*K*h*ν*)^1/2^ = 0 is the direct band gap value. The results showed that the E_g_ value for the Zn_5_Al-MMO, Zn_7_Al-MMO, and Zn_9_Al-MMO were, respectively, found to be 2.85, 2.89, and 2.87 eV, which was lower than that for pure ZnO (3.37 eV) [41]. The narrowed band gap made it possible to effectively utilize UV light energy and it was expected to improve photocatalytic activity of Zn_n_Al-MMO photocatalyst.

**Figure 5:**
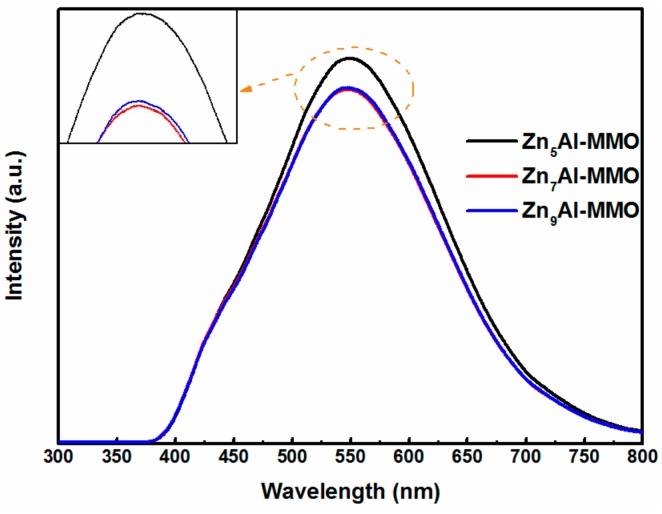
The PL spectra of Zn_n_Al-MMO composites with different Zn/Al molar ratios.

The PL spectra of Zn_n_Al-MMO nanocomposites synthesized with different Zn to Al molar ratios are shown in Figure 5. A strong emission peak was observed for all samples at ca. 550 nm and the intensity of the emission peak followed the order: Zn_5_Al-MMO > Zn_9_Al-MMO > Zn_7_Al-MMO. The PL intensity represents the recombination rate of photogenerated electrons-hole pairs. It has been established that the recombination rate of photogenerated electrons-hole pairs is lower and the photogenerated charge carriers have a prolonged lifetime as the PL intensity decreases, which is beneficial to photocatalytic performance [42]. So, it is reasonable to deduce that photocatalytic Cr(VI) reduction activity followed the order: Zn_7_Al-MMO > Zn_9_Al-MMO > Zn_5_Al-MMO.

### 3.2. Photoreduction of Aqueous Cr(VI) under UV Light

**Figure 6:**
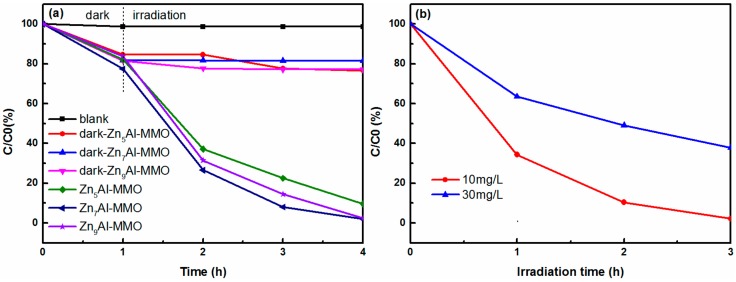
(**a**) Photoreduction of aqueous Cr(VI) using Zn_n_Al-MMO photocatalysts under UV light and (**b**) effect of initial Cr(VI) concentration on the photoreduction activity of Zn_7_Al-MMO photocatalyst.

The photocatalytic activities of Zn_n_Al-MMO nanocomposites were evaluated by the photocatalytic reduction of aqueous Cr(VI) under UV light irradiation. From Figure 6a, when the initial Cr(VI) concentration was 10 mg/L, the aqueous Cr(VI) solution was very stable under UV light in the blank experiment. In the dark adsorption experiments, the removal percentage was 23.3%, 18.5%, and 22.8% within 4 h for Zn_5_Al-MMO, Zn_7_Al-MMO, and Zn_9_Al-MMO, respectively, and the removal ratio value was almost unchanged for these three samples, indicating that the adsorption performance was independent on Zn^2+^ to Al^3+^ molar ratio of the original starting composition. Upon UV light irradiation, the Cr(VI) removal percentage was greatly enhanced within the same time intervals using Zn_n_Al-MMO samples as the photocatalyst. Zn_5_Al-MMO, Zn_7_Al-MMO and Zn_9_Al-MMO photocatalysts exhibited 90.3%, 98%, and 97.6% removal activity for aqueous Cr(VI), indicating that the Cr(VI) removal was predominated by the photocatalytic reduction process and the removal activity of the photocatalysts followed the order: Zn_7_Al-MMO > Zn_9_Al-MMO > Zn_5_Al-MMO. A pseudo first-order linear relationship was further analyzed by the plots of −ln(*C*/*C*_0_) = *k*t for Zn_n_Al-MMO, where *k* was the rate constant (h^−1^) and t was the irradiation time (h). The determined reaction-rate constants *k* were 0.69166, 1.26331, and 1.14101 h^−1^ for Zn_5_Al-MMO, Zn_7_Al-MMO, and Zn_9_Al-MMO, respectively. There was a slight difference of the reaction rates in this photocatalytic system but they were much higher than previous report [41]. During the photocatalytic progresses, the solution color from bright yellow to greenish obviously manifested the reduction of Cr(VI) to Cr(III) [41]. As mentioned in the XRD, Zn_n_Al-MMO nanocomposites were composed of ZnO and ZnAl_2_O_4_ phases, where there was a heterojunction between the interface of the composite. Higher content of Zn element of the starting materials meant more content of ZnO phase in the composites, leading to excessive amount of ZnO, which could not form heterojunction with the ZnAl_2_O_4_ counterparts. This phenomenon implied the significance of the heterojunction between ZnO and ZnAl_2_O_4_ phases of Zn_n_Al-MMO nanocomposites toward photoreduction of aqueous Cr(VI). Moreover, our hierarchically flower-like ZnO/ZnAl_2_O_4_ composite is a three-dimensional micro- and nanometer assembly with highly porous structure and high specific surface area. Usually, hierarchical architecture contributes to the generation and migration of photo-carrier and provides more sites for pollutant and incident light adsorption. In combination with the structure analysis, it is easy to understand that Zn_7_Al-MMO with loosely 3D flower-like hierarchical structure exhibited higher photocatalytical activity toward the reduction of aqueous Cr(VI).

Figure 6b shows the effect of initial Cr(VI) concentration on the photocatalytic activity of Zn_7_Al-MMO photocatalysts. When initial Cr(VI) concentrations were 10 and 30 mg/L, the photocatalytic removal percentage of aqueous Cr(VI) was almost 98% and 63% in three hours of irradiation time, respectively. Increasing the initial Cr(VI) concentration enhanced the amount of Cr(VI) adsorbed on the surface of the catalyst while the photoelectron-hole pairs generated by the photo-excited catalyst didn’t increase due to the unchanged amount of catalyst, so the effective Cr(VI) concentration reduced was relatively decreased. Moreover, increased absorption of UV by the solution occurred since higher Cr(VI) solution could absorb more light in the UV-Vis range, leading to low light absorption of the photocatalyst. Additionally, with the increasing concentration of aqueous Cr(VI), the color of the solution became darker, which was not conducive to the absorption of UV light photons by the catalyst, thus reducing the efficiency of photoreduction of aqueous Cr(VI) [43]. While considering that the Cr(VI) concentration commonly used in the ZnO or TiO_2_-based photoreduction experiments tended to be low [44,45,46], the photoreduction activity while using as-prepared Zn_7_Al-MMO photocatalyst was actually quite fast.

**Figure 7:**
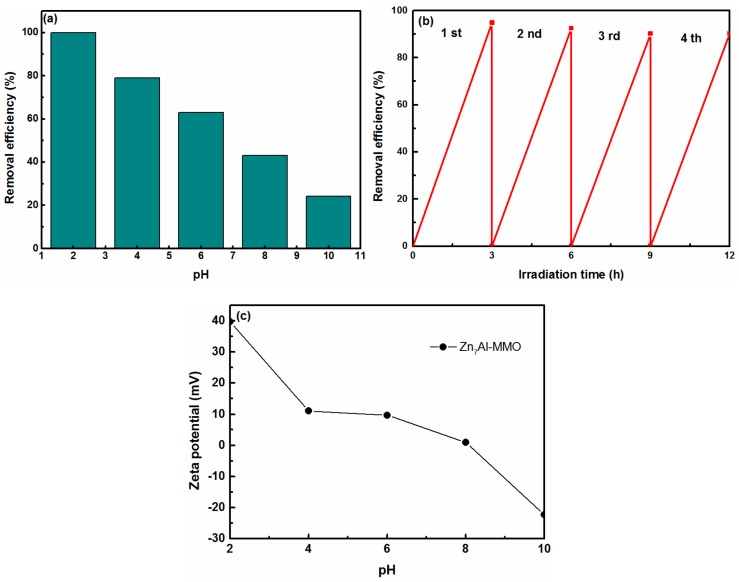
Effect of (**a**) initial pH and (**b**) cycling tests of photocatalytic activity of the Zn_7_Al-MMO for the photoreduction of Cr(VI). (**c**) Zeta potential of Zn_7_Al-MMO in different initial pH solution.

The effect of differently initial pH on the photoreduction of Cr(VI) while using Zn_7_Al-MMO photocatalyst was evaluated with the Cr(VI) concentration of 30 mg/L. The pH of solution is one of the most important influence factors in the photocatalytic process. From Figure 7a, the photoreduction removal percentage of Cr(VI) was increased from 63 to 100% within 3 h’s irradiation with cutting down the initial pH values from 6.01 (the pH of the original solution) to 2, whereas the removal percentage of Cr(VI) was only 79%, 43%, and 24% at pH = 4, 8, and 10 under the same conditions, respectively. The results that the solution in acidic conditions has higher photoreduction efficiency for Cr(VI) than in alkaline conditions was in agreement with the report [47]. In an acidic solution, the surface of the photocatalyst may be highly protonated, resulting in a strong electrostatic attraction to the main species of chromium anions, HCrO^4−^ or Cr_2_O_7_^2−^, in a low pH solution [48]. At low pH, the reduction potential of the couple Cr^6+^/Cr^3+^ becomes more positive. Additionally, Cr(OH)_3_ is deposited on the surface of the photocatalyst at high pH to inhibit the reduction of Cr(VI) [49]. Meanwhile, Zn_7_Al-MMO exhibits different zeta potentials in different pH solutions (Figure 7c). The pH is especially crucial because it affects the surface charge of the adsorbents [50]. From Figure 7c, as the pH of the solution gradually increased from 2 to 10, the surface charge of Zn_7_Al-MMO decreased, from +39.73 to −22.3. At low pH solution, the surface of photocatalyst was positively charged, attracting HCrO^4−^ and Cr_2_O_7_^2−^ plasmas, thus promoting the Cr(VI) reduction. Conversely, in high pH solution, the surface of photocatalyst was much negatively charged, repelling chromium ions in the form of CrO_4_^2−^, thus depressing the reduction rate of Cr(VI).

The re-use of catalysts is very important for practical application. However, according to investigations, there is no research on the long term stability of ZnO/ZnAl_2_O_4_ for Cr(VI) photoreduction [34]. Repetitive reduction of Cr(VI) by Zn_7_Al-MMO nanocomposite under UV light irradiation was conducted to study their long-term stability and the results are shown in Figure 7b. In this experiment, we used an initial Cr(VI) concentration of 10 mg/L. After each cycle, the photocatalyst was separated from the solution by centrifugation and then rinsed with distilled water for next runs. From Figure 7b, it can be observed that the removal percentage of Cr(VI) still reached 90% and the loss of efficiency was only 5% after fourth cycle, which could be ascribed to the loss of catalyst during centrifugation after each cycle. These results suggested that the 3D sphere-like hierarchical ZnO/ZnAl_2_O_4_ nanocomposite exhibited long-standing high photoreduction activity in recycles, and thus it could have potential application prospects in the water environmental restoration. 

**Figure 8:**
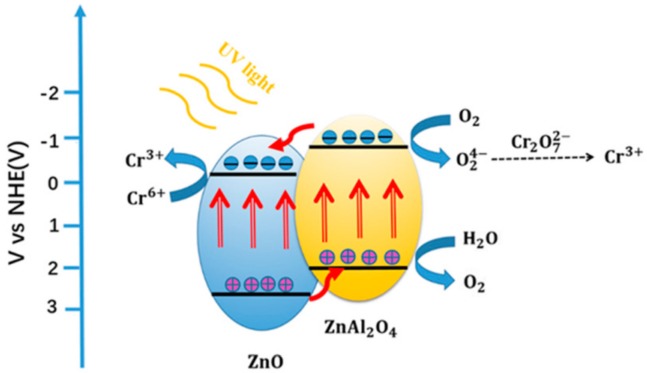
Schematic diagram of electron-hole separation and transport mechanism of photoreduction of Cr(VI) while using ZnAl-MMO photocatalyst under UV light irradiation.

Figure 8 shows the synergistic mechanism between ZnO and ZnAl_2_O_4_ phases in high temperature calcined product Zn_n_Al-MMO for photoreduction of Cr(VI). The ZnO phase and the ZnAl_2_O_4_ phase absorb photon energy under UV light irradiation. The valence band (VB) electron is excited to transition to the conduction band (CB) while leaving holes in VB to form photogenerated electron-hole pairs. The VB and CB potential of ZnO are +2.89 eV and −0.31 eV and those of ZnAl_2_O_4_ are +2.66 eV and −1.14 eV, respectively. Due to the CB potential of ZnAl_2_O_4_ being lower than that of ZnO, the electron at the bottom of CB of ZnAl_2_O_4_ is transferred to the surface of ZnO. While the VB potential of ZnO is higher than that of ZnAl_2_O_4_, the hole at the top of VB of ZnO is migrated to ZnAl_2_O_4_. The well-formed band structure between ZnO and ZnAl_2_O_4_ phases facilitates the migration of photogenerated carriers, thereby effectively suppressing the recombination of photogenerated electrons and holes. Since the CB potential of ZnO is more negative than the Cr(VI) standard potential of E^θ^(Cr_2_O_7_^2−^/Cr^3+^) = +1.33 V, photogenerated electrons reduce Cr^6+^ to Cr^3+^ (Equation (1)). Meanwhile, due to the more positive VB position of ZnAl_2_O_4_ than the oxidation potential of E^θ^(H_2_O/O_2_) = +1.23 V, the holes oxidize H_2_O to form O_2_ (Equation (2)). The consumption of the holes greatly reduces the rate of recombination of photogenerated carriers, enhancing the photocatalytic activity of ZnAl-MMO nanocomposite towards Cr(VI) reduction.

Cr_2_O_7_^2−^ + 14H^+^ + 6e → 2Cr^3+^ + 7H_2_O (1)

2H_2_O + 4h^+^ → O_2_ + 4H^+^ (2)

## 4. Conclusions

In conclusion, hierarchical ZnO/ZnAl_2_O_4_ composites with 3D sphere-like nanostructures synthesized through a simple hydrothermal process and subsequent high-temperature calcination. The molar ratio of the Zn/Al metallic cations in the starting mixture played a key role in the morphological transformation of the resulting products from cubic aggregates, hierarchically flower-like spheres to porous microspheres and all the building blocks of the composites exhibited a heterojunction nanostructure between ZnO and ZnAl_2_O_4_ phases. ZnO/ZnAl_2_O_4_ composite showed outstanding photocatalytic activity towards aqueous Cr(VI) reduction under UV light irradiation. The excellent photoreduction performance of the ZnO/ZnAl_2_O_4_ nanocomposite was attributed to 3D sphere-like hierarchical structure and well-formed heterojunctions within the nanocomposite.
